# Lipoprotein Lpp regulates the mechanical properties of the *E. coli* cell envelope

**DOI:** 10.1038/s41467-020-15489-1

**Published:** 2020-04-14

**Authors:** Marion Mathelié-Guinlet, Abir T. Asmar, Jean-François Collet, Yves F. Dufrêne

**Affiliations:** 1 Institute of Life Sciences, UCLouvain, Croix du Sud, 4-5, bte L7.07.06, B-1348 Louvain-la-Neuve, Belgium; 2 de Duve Institute, UCLouvain, Brussels, Belgium; 3 Walloon Excellence in Life sciences and Biotechnology (WELBIO), Wavre, Belgium

**Keywords:** Membrane biophysics, Cellular microbiology, Bacteriology

## Abstract

The mechanical properties of the cell envelope in Gram-negative bacteria are controlled by the peptidoglycan, the outer membrane, and the proteins interacting with both layers. In *Escherichia coli*, the lipoprotein Lpp provides the only covalent crosslink between the outer membrane and the peptidoglycan. Here, we use single-cell atomic force microscopy and genetically engineered strains to study the contribution of Lpp to cell envelope mechanics. We show that Lpp contributes to cell envelope stiffness in two ways: by covalently connecting the outer membrane to the peptidoglycan, and by controlling the width of the periplasmic space. Furthermore, mutations affecting Lpp function substantially increase bacterial susceptibility to the antibiotic vancomycin, indicating that Lpp-dependent effects can affect antibacterial drug efficacy.

## Abstract (web summary)

Lipoprotein Lpp provides a covalent crosslink between the outer membrane and the peptidoglycan in *E. coli*. Here, the authors use atomic force microscopy to show that Lpp contributes to cell envelope stiffness by covalently connecting the two layers and by controlling the width of the periplasmic space.

## Introduction

**Fig. 1:**
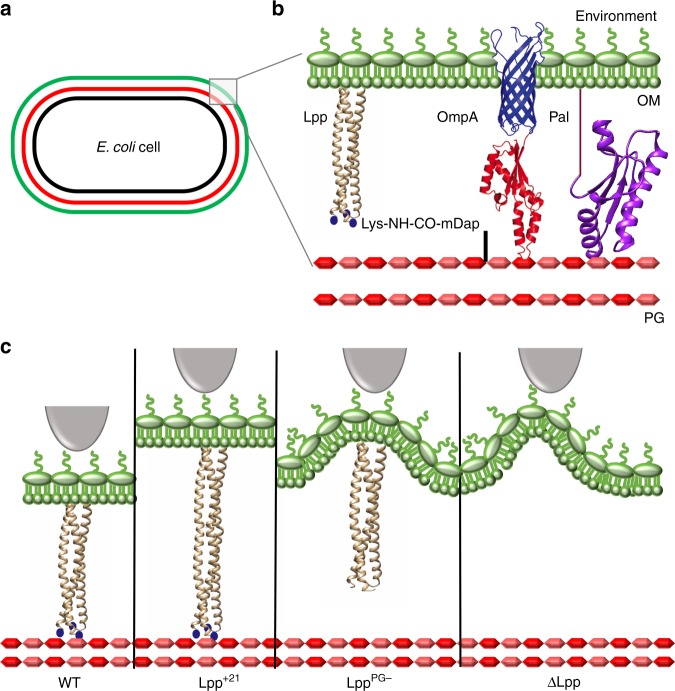
Studying the mechanical properties of the *E. coli* cell envelope by means of mutants altered in their Lpp proteins. **a** Scheme of the *E. coli* cell showing its multilayered envelope composed of an inner (IM, black) and an outer (OM, green) membranes separated by the periplasm containing a peptidoglycan layer (PG, red). **b** Closer view of the envelope emphasizing the presence of proteins from the OM connecting it to the PG. The lipoprotein Lpp is the only OM lipoprotein covalently crosslinked to PG while being inserted in the OM by a lipid moiety attached to an N-terminal cysteine. Lpp is drawn as a trimeric α-helical coiled coil based on its crystal structure (PDB: 1EQ7). C-terminal lysines are added as blue dots as they were lacking in the abovementioned crystal structure. Pal is another OM lipoprotein that interacts noncovalently with the PG: crystal structure (PDB: 1OAP). OmpA is a β-barrel protein composed of a β-barrel domain inserted in the OM (predicted structure represented in blue) and a periplasmic domain that interacts noncovalently with the PG (represented in red: PDB: 4ERH). **c** Scheme depicting four representative Lpp mutant strains studied in this paper. Lpp variants representation was adapted from its crystal structure (PDB: 1EQ7). WT wild type, Lys lysine, mDap diaminopimelic acid.

Bacteria are surrounded by mechanically strong cell walls that fulfill several important functions, including defining cell shape, determining growth, and division, resisting turgor pressure, mediating cellular interactions, and protecting cells from hostile environments and drugs^1^. In Gram-negative bacteria, such as *Escherichia coli*, the cell envelope is made up of an inner membrane (IM; a classical phospholipid bilayer around the cytoplasm) and an outer membrane (OM; an asymmetric structure with phospholipids in the inner leaflet and lipopolysaccharides in the outer leaflet) separated by an aqueous environment, the periplasm (Fig. 1a)^1^. This later compartment contains peptidoglycan (PG), a polymer of glycan strands cross-linked by short peptides that provides shape and rigidity to the cell^2^. Considerable efforts have been dedicated to understand how the cell envelope and its environment affect the mechanical behavior of bacteria and, in turn, how such properties allow them to fight against external stress^3^. It has been recently shown that the stiffness of *E. coli* is defined not only by the PG but also by the OM, and that mechanical loads are balanced between these structures^4^. In addition, there is evidence that cell mechanics may also depend on protein interconnections between these two layers^4^. Among these proteins, the alpha-helical lipoprotein Lpp (also known as Braun’s lipoprotein) is numerically the most abundant protein in *E. coli*, with more than 1 million copies per cell^5,6^. Lpp provides the only covalent crosslink between the OM and the PG through its N-terminus and C-terminus, respectively (Fig. 1b), tethering the OM to the PG and determining the size of the periplasm^7,8^. While the OM–PG covalent bridge and the length of Lpp are known to be of importance for the transmission of stress signals from the OM to the IM^7^ and for flagellum assembly^8^, we currently know very little about the mechanical role(s) of Lpp and its (their) physiological implication(s).

Atomic force microscopy (AFM) has emerged as a valuable multifunctional technique to investigate the structure, properties, and functions of microbial cells^9^. In mechanobiology, AFM-based indentation experiments have enabled to quantify the mechanical properties of whole bacterial cells and isolated cell walls^10^. Here, AFM was used to capture the structural and mechanical properties of *E. coli*, with the aim to answer the following questions: does Lpp play a role in regulating cell envelope mechanics; is the mechanical function of Lpp primarily controlled through the physical connection between OM and PG, and/or through modulation of the periplasmic width; does Lpp-dependent cell mechanics influence the sensitivity to antibiotics? To this end, we compared the behavior of wild-type (WT) bacteria with that of bacterial strains with Lpp functional mutations, i.e., Lpp elongated by 7, 14, or 21 residues (hereafter Lpp^+7^, Lpp^+14^, Lpp^+21^, respectively), Lpp lacking the covalent connection to the PG (Lpp^PG−^ and Lpp^+21,PG−^), and cells lacking Lpp (ΔLpp) (Fig. 1c). The results show that the Lpp-dependent OM–PG connection plays a key role in controlling the stiffness of the cell envelope and its sensitivity to drugs, with the OM-to-IM separation distance and the Lpp-PG covalent link being of particular importance.

## Results

### Lpp influences cell shape

**Fig. 2:**
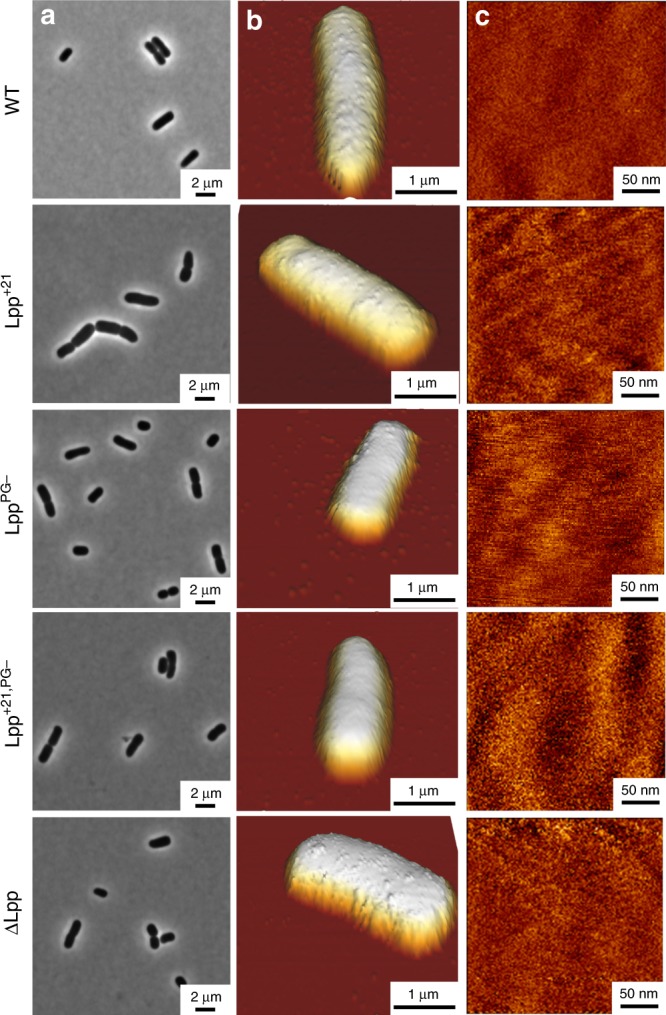
Effect of Lpp mutations on cell morphology and surface ultrastructure. **a** Representative phase-contrast images reveal cell morphology of *E. coli* cells on agarose pads. **b** AFM height images in PBS showing *E. coli* cells immobilized on PEI-coated coverslips (color scale: 1.2 µm). **c** High-resolution height images recorded on top of the cells shown in (**b**) (color scale: 10 nm). These images are representative of at least three independent experiments.

It has long been considered that the shape of Gram-negative bacteria and their ability to sustain turgor pressure are mainly defined by the PG layer. However, recent studies have indicated that the OM and the tight protein interconnections between this membrane and the PG are also involved^4^. We therefore first sought to investigate whether functional mutations in Lpp induce morphological and structural changes in *E. coli*. Bacteria growing in exponential phase were immobilized on PEI-coated coverslips and imaged in PBS buffer, without any chemical fixation to preserve their native structure (Fig. 2). Phase microscopy (Fig. 2a) and AFM low-resolution 3D height images (Fig. 2b) revealed the expected rod shape of *E. coli* for representative cells of each strain (WT, Lpp^+21^, Lpp^PG−^, Lpp^+21,PG−^ ΔLpp). In addition, high-resolution images of the cell surface did not feature any changes between the strains (Fig. 2c). Smooth surface morphologies were observed, with a r.m.s. roughness of 2.1 ± 1.4 nm for all strains (mean from a total of 53 images), implying that Lpp mutations did not induce any substantial changes in cell surface ultrastructure. Note however that considering the variability inherent to cells in the exponential phase, fuzzy features were sometimes observed on all strains, that we attribute to the interaction between the AFM tip and LPS.

**Fig. 3:**
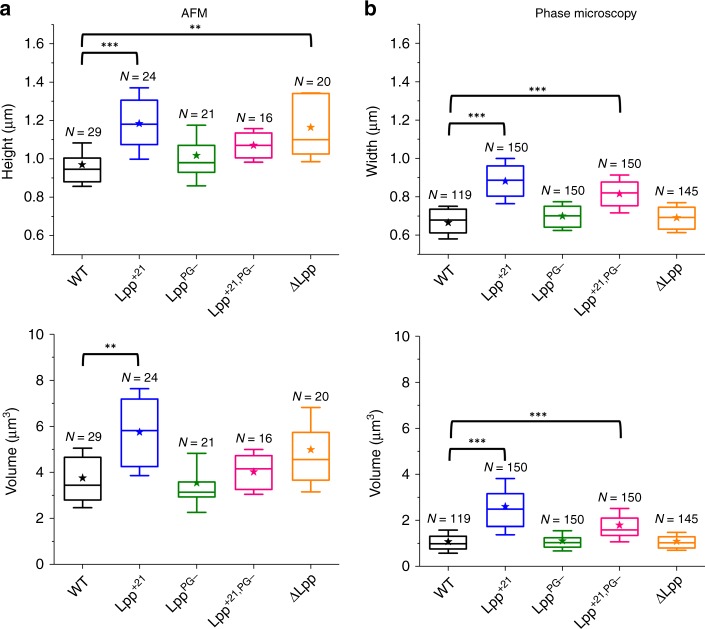
Effect of Lpp mutations on *E. coli* dimensions. Box plots reporting the height/width and volume of each bacterial strain estimated from (**a**) AFM images and (**b**) phase microscopy cliches. Shown here are the mean values (star), the median, the 25 and 75% quartiles (boxes), and the standard deviation (whiskers) obtained from N independent cells over at least three independent experiments. **P ≤ 0.001 and ***P ≤ 0.0001. Source data are provided as a Source Data file.

Statistical analysis of *E. coli* dimensions obtained by AFM (Fig. 3a) suggests that increasing the length of Lpp by 21 residues or removing the protein induces an increase in height, from 1.0 ± 0.1 µm for WT (mean value and SD from *n* = 29 cells) to 1.1 ± 0.1 µm for Lpp^+21,PG−^ (*n* = 16) and 1.2 ± 0.2 µm for Lpp^+21^ (*n* = 24) and ΔLpp (*n* = 20). However, this phenomenon is not necessarily correlated with a significant change in cell length, and therefore in cell volume (Fig. 3a, Supplementary Fig. 1). To further analyze cell sizes at the population level, we captured hundreds of cells using optical phase microscopy, especially for cells expressing a longer version of Lpp (Lpp^+21^ and Lpp^+21,PG−^) (Fig. 3b, Supplementary Fig. 1). In contrast, cells expressing Lpp variants with moderately increased lengths (Lpp^+7^ and Lpp^+14^) or a version of Lpp not covalently linked to PG (Lpp^PG−^) did not exhibit such an increase in cell height (0.9 ± 0.1 µm, *n* = 23 cells for Lpp^+7^; 1.0 ± 0.1 µm, *n* = 20 cells for Lpp^+14^, and 1.0 ± 0.2 µm, *n* = 21 cells for Lpp^PG−^) (Fig. 3, Supplementary Fig. 2). Of note, these modifications could not be attributed to the time spent in PBS solvent as no significant differences were observed over time when following WT and Lpp^+21^ cells (Supplementary Fig. 3A).

### Lpp plays a major role in defining the cell envelope mechanics

**Fig. 4:**
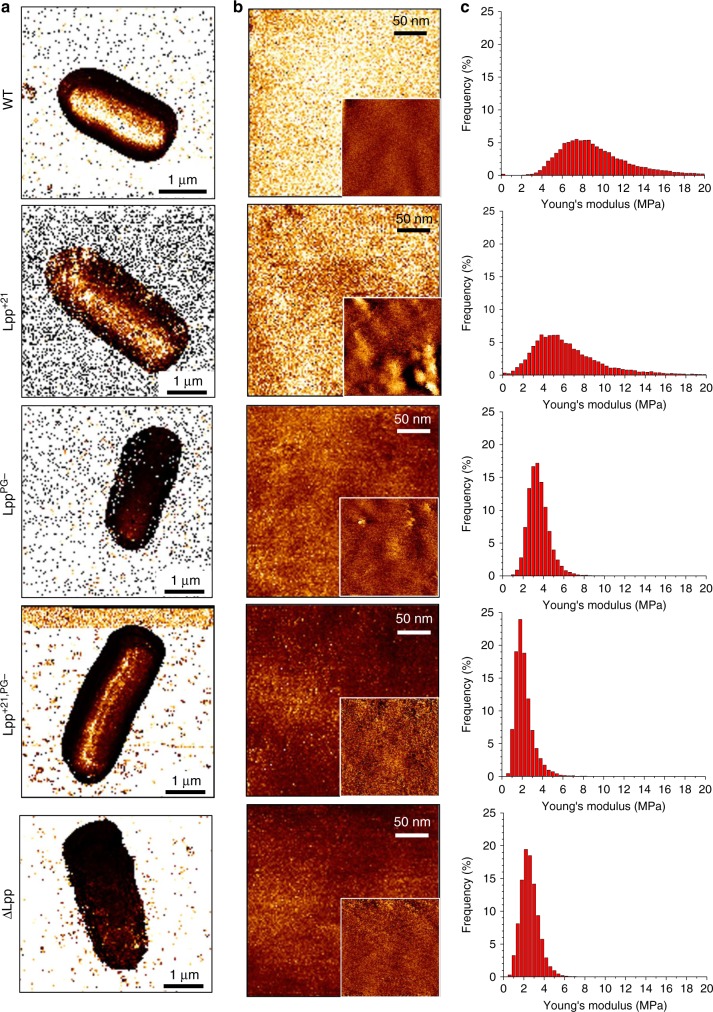
Multiparametric imaging of *E. coli* WT and Lpp mutants. **a** Elasticity maps in PBS of representative cells from the main *E. coli* strains (QI mode, 128 × 128 curves, color scale: 10 MPa). **b** High-resolution elasticity maps recorded on top of the cell (color scale: 10 MPa) along with corresponding height images (insets, color scale: 10 nm). **c** Distribution of Young’s modulus values corresponding to the elasticity maps in **b**. These images are representative of at least three independent experiments. Source data are provided as a Source Data file.

The Lpp mutations investigated here have been previously shown to drastically change the permeability and integrity of the *E. coli* cell envelope^11–13^. We therefore hypothesized that these mutations may also alter the mechanical properties of the cells. We mapped the structure and elasticity of single live cells at high spatio-temporal resolution, using multiparametric quantitative imaging (QI)^14,15^ (Fig. 4). The stiffness (or tensile elasticity) of a sample is classically defined by its Young’s modulus, *E*^16^. Low (Fig. 4a) and high (Fig. 4b) resolution elasticity (*E*) maps did not feature any major heterogeneities, suggesting that cell surfaces are physically homogeneous. Consistent with the above data, correlated high-resolution height images of the cells showed a smooth, featureless surface morphology (Fig. 4b, insets). Notably, *E* values for the three single mutants (3.2 ± 2.6 MPa (*n* = 7), 2.6 ± 2.5 MPa (*n* = 8), and 2.4 ± 1.4 MPa (*n* = 4) for Lpp^+21^, Lpp^PG−^, and ΔLpp, respectively) were substantially lower than for the WT (5.2 ± 5.2 MPa (*n* = 7)), indicating that either removing Lpp from the OM or altering its function induce a softening of the cell envelope (Fig. 4c). Interestingly, the mechanical features of the different Lpp length variants tested were similar (3.4 ± 1.8 MPa (*n* = 3), 3.0 ± 1.8 MPa (*n* = 6), and 3.2 ± 2.6 MPa (*n* = 7) for Lpp^+7^, Lpp^+14^, and Lpp^+21^, respectively) (Supplementary Fig. 4A). In contrast, increasing the length of the Lpp variant that cannot be crosslinked to the PG Lpp^+21,PG−^ led to a further softening of the envelope (2.0 ± 0.2 MPa (*n* = 3)) (Fig. 4).

**Fig. 5:**
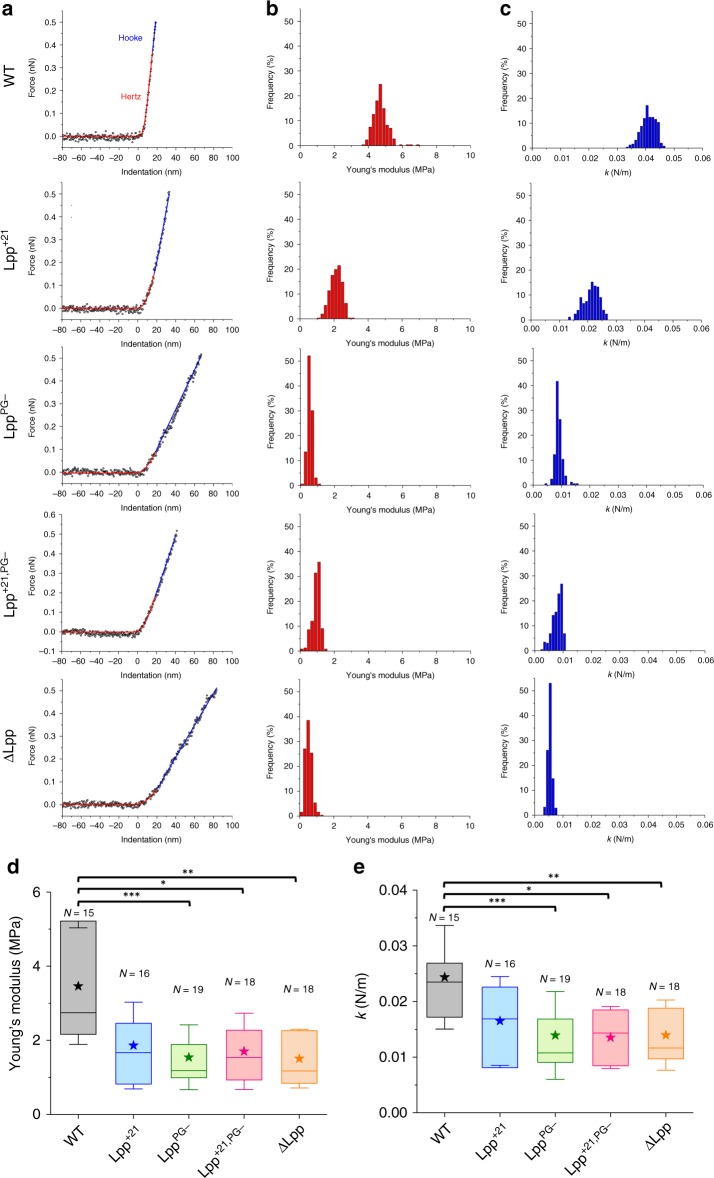
Deciphering the molecular basis of Lpp-dependent cell envelope mechanics. **a** Representative force-indentation curves with theoretical fits used to extract the Young’s modulus (Hertz fit, red) and the bacterial spring constant (Hooke fit, blue) for the different *E. coli* strains. Distribution of Young’s modulus (**b**) and spring constant (**c**) values obtained by force–volume measurements across the surface of one representative cell (16 × 16 curves, 250 × 250 nm²). **d**, **e** Statistical analysis performed for each strain, showing the Lpp-dependent softening of *E. coli* strains. Shown here are the mean values (stars), the median, the 25 and 75% quartiles (boxes), and the standard deviation (whiskers) obtained from N independent cells over at least three independent experiments. *P ≤ 0.005, **P ≤ 0.001, and ***P ≤ 0.0001. Source data are provided as a Source Data file.

To understand the molecular basis of the Lpp-induced changes in cell envelope mechanics, we recorded spatially resolved force curves on top of the cells using force–volume (FV) imaging^17,18^. The curves were converted into force vs indentation curves and further analyzed to get detailed information on sample stiffness. Representative data obtained on the different strains are presented in Fig. 5a. As expected for Gram-negative bacteria^19^, the curves featured two regions, i.e., a nonlinear regime at low forces reflecting cell wall elasticity followed by a linear regime at high forces associated with turgor pressure. From these regimes, we assessed the bacterial Young’s modulus, *E*, and the bacterial spring constant, *k*_b_^19–21^. For WT cells, analysis of the curves using the Hertz model (Fig. 5b, d) yielded *E* of 3.5 ± 1.6 MPa (mean ± SD, *n* = 15 cells), in agreement with previously published values ranging from 1 to few MPa on *E. coli*^22–24^. The *k*_b_ values were found to be 0.02 ± 0.01 N m^−1^ (Fig. 5c, e), which is in the range of values reported for *E. coli* (0.04 N m^−1^)^25,26^ and for the two other Gram-negative bacteria *Shewanella putrefaciens* (0.02–0.05 N m^−1^)^19^ and *Pseudomonas aeruginosa* (0.02 N m^−1^)^27^. We note that similar trends were obtained using the FV and QI modes, the later yielding larger values, which we believe results from the much higher speed of QI^28^.

As the stiff cell wall of *E. coli* defines important cellular functions and given that Lpp has proved to be involved in cell permeability^11,29–31^, we postulated that this lipoprotein may impact the mechanical strength of the cell envelope. Figure 5 shows that Lpp^PG−^ and ΔLpp strains were much softer than the WT, as shown by the shift toward lower *E* values (1.5 ± 0.9 MPa (*n* = 19) and 1.5 ± 0.8 MPa (*n* = 18), respectively) and *k*_b_ values (0.01 ± 0.01 N m^−1^ for Lpp^PG−^ and ΔLpp, respectively). Thus, ΔLpp cells are twice softer than WT cells, which agrees well with earlier reports^4,32^. Interestingly, cells expressing Lpp^PG−^ or lacking Lpp (∆Lpp) show a similar behavior, which suggests that the covalent link between Lpp and PG is the main factor contributing to cell stiffness, rather than the presence of the protein itself.

Elongation of Lpp alters signaling processes, revealing the physiological importance of the size of the periplasm^7^. We therefore asked whether Lpp lengthening alters the cell envelope mechanics as observed for ΔLpp and Lpp^PG−^. As shown in Fig. 5, Lpp^+21^ cells displayed softer properties than WT cells, with average values of *E* = 1.9 ± 1.2 MPa (*n* = 16) and *k*_b_ = 0.02 ± 0.01 N m^−1^. Moderately increasing the length of Lpp had the same impact: mutant cells were softer than WT, in a way similar to Lpp^+21^: *E* = 2.2 ± 1.0 MPa (*n* = 18), *E* = 1.8 ± 0.9 MPa (*n* = 19), and *k*_b_ = 0.02 ± 0.01 N m^−1^ for Lpp^+7^ and Lpp^+14^, respectively, (Supplementary Fig. 4B–E). This suggests that the length of Lpp is optimal for *E. coli* mechanical properties, and that increasing Lpp’s length even by seven residues (around 1 nm in length) interferes with the mechanical role of Lpp in the cell envelope. Analysis of the mutant both lacking the connection to the PG and expressing a longer version of Lpp did not show a substantial decrease in the mechanical properties of *E. coli* as compared with Lpp^PG−^ (*E* = 1.7 ± 1.0 MPa (*n* = 18) and *k*_b_ = 0.01 ± 0.01 N m^−1^ for Lpp^+21,PG−^) (Fig. 5).

Given those close values, and knowing that PBS has no influence on cell mechanics over time (Supplementary Fig. 3B), we can conclude that the property of Lpp that plays a dominant role in the final elasticity of *E. coli* cells is its physical connection to the PG. However, the length of Lpp also controlling cell softening, but more moderately, implies that Lpp might regulate cell envelope mechanics by tightly controlling its molecular length. These mechanical differences are not directly correlated with the potential morphological impact of some Lpp mutations (Supplementary Fig. 5).

### Lpp-dependent cell softening increases antibiotics sensitivity

**Fig. 6:**
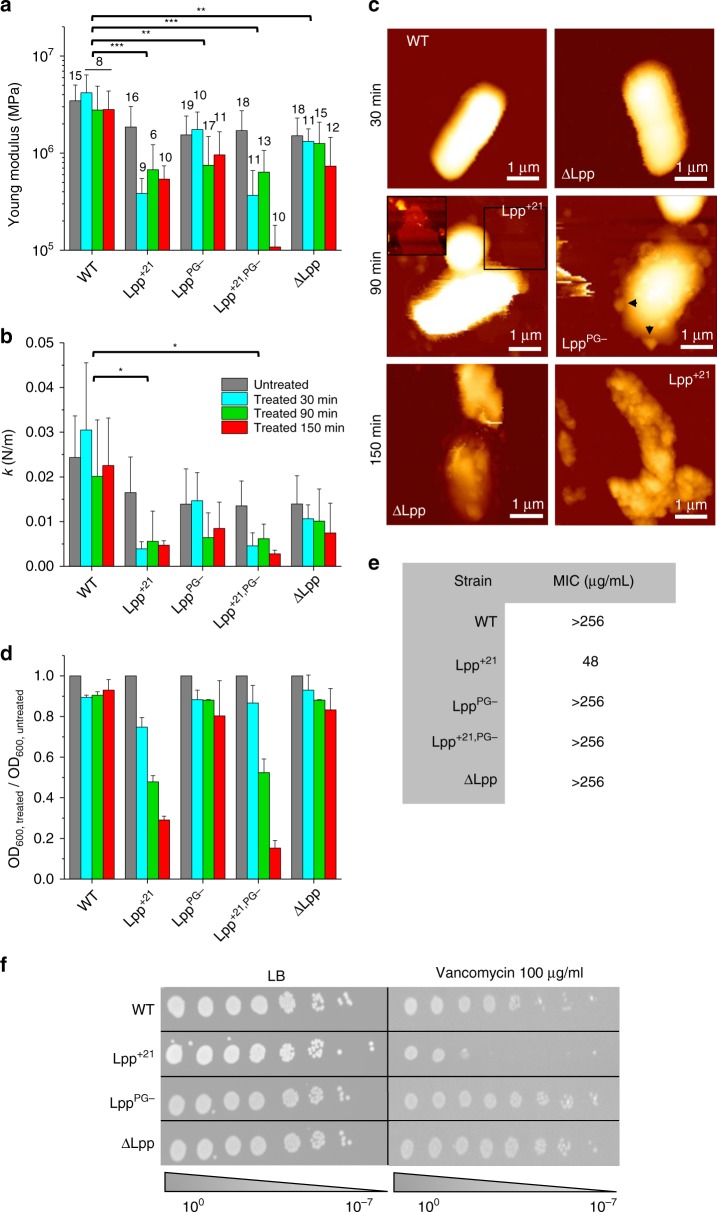
Clinical relevance of Lpp-dependent cell mechanics: Lpp mutations increase susceptibility of the cells to vancomycin. Time-dependent susceptibility of each bacterial strain toward vancomycin, as expressed by the change in the Young’s modulus (**a**) and the cell stiffness (**b**). Standard deviations are obtained from N independent cells over at least three independent experiments (data are presented as mean ± SD). **c** AFM height images (color scale: 1.2 µm) of *E. coli* strains after vancomycin treatment (100 µg/mL). The inset for Lpp^+21^ is a closer view corresponding to the black square (1.5 µm × 1.5 µm) showing a lysed cell. Black arrows show blebs. These images are representative of at least three independent experiments. **d** Evolution of the OD_600_ of each strain while being treated with vancomycin (data are presented as mean ± SD). **e** Table summarizing MIC values for different *E. coli* strains. Values are representative of four biological replicates. **f** Vancomycin sensitivity spotting assay. Cells were serially diluted and spotted overnight on LB agar plates containing vancomycin where indicated. Data shown are representative of three biological replicates. WT wild type, LB Luria-Broth. *P ≤ 0.005, **P ≤ 0.001, and ***P ≤ 0.0001. Source data are provided as a Source Data file.

Finally, given the role of Lpp in maintaining the permeability features of the cell envelope, we wondered if Lpp mutations correlated with higher susceptibility to vancomycin, an antibiotic that binds specifically to PG precursors thus preventing their incorporation in the cell wall. Vancomycin is normally known to be unable to cross the OM in Gram-negative bacteria such as *E. coli*, unless the OM is defective^33^. We analyzed the structural and mechanical properties of bacteria pretreated with vancomycin during 30 min (Fig. 6a–c and Supplementary Fig. 6) at a concentration of 100 µg mL^−1^, which is below the MIC for most of the strains (Fig. 6e). For this study, considering the similar behavior of cells expressing Lpp^+7^, Lpp^+14^, and Lpp^+21^ before vancomycin treatment, we decided to focus on the longest Lpp variant, i.e., Lpp^+21^. WT, Lpp^PG−^, and ΔLpp cells were barely affected by the treatment with vancomycin, both from a mechanical and morphological perspective: they remained rod shaped, displaying homogeneous ultrastructure, and conserved an elastic behavior similar to that previously shown for untreated cells. In contrast, when treated with vancomycin, cells expressing Lpp^+21^ and Lpp^+21,PG−^ exhibited lower Young’s modulus and stiffness (Fig. 6a, b). They also showed morphological damage (decrease in volume, change in shape/ultrastructure; Supplementary Fig. 6), potentially leading to cell lysis.

We further studied the influence of the time of treatment (30, 90, and 150 min) on the mechanics of the different mutants (Fig. 6a–c and Supplementary Fig. 6). Even after 150 min of treatment, WT cells remained mostly unaffected, keeping their initial mechanics and morphology, confirming the inability of vancomycin to cross the OM when its integrity is preserved. A complete deletion of Lpp led to a relative decrease in (*E* and *k* values) but only after 150 min of treatment; this softening was not associated, in the majority of cases, with morphological damage. Noteworthy, some cells featured small globular protrusions, 100–150 nm in height (Supplementary Fig. 6, arrows), which we attribute to OM vesicles^34^. Cells lacking the PG covalent connection (Lpp^PG−^) reacted faster to the treatment: after 90 min, they became significantly softer and drastically modified their shape: well-defined cell edges were no longer observed, cell surface was extremely rough, and some cells were even broken. However, mutants expressing a longer version of Lpp (Lpp^+21^ and Lpp^+21,PG−^) were the most affected, with a majority of cells showing partial or complete lysis, some adopting a more spherical shape or blebbing like observed with the ΔLpp mutant.

Morphological and mechanical changes were found to correlate well with OD measurements (Fig. 6d). By comparing the OD of untreated and treated cells at the same time point (30, 90, and 150 min), one can have a clue on the amount of lysis encountered in the studied suspension. This experiment showed that (i) WT cells were hardly affected by vancomycin, whatever the time of treatment, (ii) Lpp^PG−^ and ΔLpp cells were moderately affected after 150 min of treatment (decrease of ~30% in OD), and (iii) Lpp^+21^ and Lpp^+21,PG−^ showed a larger decrease in OD with time of antibiotic treatment, suggesting an increased proportion of lysis. Finally our results were also in agreement with antibiotic spotting assays (Fig. 6f), showing qualitatively that, below the minimal inhibitory concentration (Fig. 6e), while most strains remained largely unaffected, the Lpp^+21^ mutant showed the highest sensitivity to vancomycin. This highlights the robustness of AFM to visualize cell surface damages on the nanoscale, which were not observed on a larger scale in spotting assays. In summary, our results show that the nature of the OM–PG connection (molecular length of Lpp and covalent bridge) plays an essential role in controlling the mechanics of *E. coli* cells and their sensitivity to antibiotics, probably via the permeability of the OM, the presence or absence of Lpp per se playing little role in drug susceptibility.

## Discussion

The mechanical properties of bacteria are critical for cellular function, physiology, and pathogenicity^16,35^. Because of the stiff architecture of their multilayered cell envelope, Gram-negative bacterial pathogens are difficult to treat, and therefore represent a serious threat for human health. It has long been considered that bacterial cell mechanics primarily relies on PG, which forms a rather stiff and compact layer targeted by various antibiotics^36,37^. Recently, however, evidence has accumulated that other cell envelope components and structures also contribute to cellular mechanics, i.e., the OM and its connection to PG in the case of Gram-negative bacteria^4^. As Lpp is numerically the most abundant protein in *E. coli*, it is tempting to speculate that it plays multiple functional roles. Accordingly, loss of Lpp favors vesicle formation, decreases membrane integrity, reduces virulence, and prevents cells to sense and respond to stress^38^. Despite the crucial physiological functions of Lpp, little is currently known about its contribution to cell mechanics. We have shown that the structural and chemical nature of the Lpp-dependent OM–PG connection—i.e., molecular length and covalent bonding—is crucial in controlling the stiffness of *E. coli* and its sensitivity to vancomycin. Our results show promise for the design of innovative antibacterial drugs targeting the molecular machineries that stabilize the OM and PG layers.

Our first finding is that Lpp might play a role in controlling the three-dimensional size of *E. coli* cells. Whereas Lpp mutations have no effect on the fine surface architecture of the bacteria, they substantially alter cell height, and they do so to different extents. In particular, AFM and phase microscopy showed that elongation of Lpp by adding 21 residues caused a significant increase in cell size, while a moderate increase of the length of Lpp (Lpp^+7^ or Lpp^+14^) or disruption of the PG covalent cross-link (Lpp^PG−^) had no effect. Such morphological differences are not resulting from different growth rates, because (i) all strains were shown to grow similarly until stationary phase (Supplementary Fig. 7A) and (ii) the increased height of Lpp^+21^ cells compared with the WT was shown in early, mid-exponential, and stationary phases (Supplementary Fig. 7B). We cannot exclude that the changes in cell shape observed in the Lpp mutants result from a potentially different behavior of these cells when exposed to osmolarity changes (the cells are transferred from a rich (LB) to a poor medium (PBS, for AFM purposes)). However, the influence of turgor pressure on cell shape is still a matter of controversy (some claiming a crucial role in cell growth^39,40^, while others suggesting a minor impact^41^), which makes difficult further interpretation of our data. It is intriguing that cell size does not change in cells expressing Lpp^+7^, Lpp^+14^, or Lpp^PG−^. However, here it is possible that envelope proteins such as the lipoprotein Pal and the β-barrel OmpA^11,42^, which confer noncovalent OM–PG crosslinking, partially compensate for the loss of Lpp periplasmic spanners, sustaining WT cell size^7^. This mechanism does not function when cells express Lpp^+21^ because this longer version of Lpp is likely to override Pal and OmpA, imposing a larger periplasm, as also suggested by the more moderate increase in cell volume for Lpp^+21,PG−^ cells. Clearly, Lpp mutations could also induce defects in the assembly machineries of the envelope, which might cause morphological alterations in the three layers of this compartment. Thus, understanding at the molecular level how the different types of Lpp mutations translate into morphological changes will require further investigation on Lpp itself and on its different functional roles. Yet, we speculate that this massively abundant protein has additional, yet-to-be identified functions in the cell envelope that could be impaired either by increasing the length of the protein or by preventing its covalent attachment to the PG. For instance, PG-bound Lpp has been shown to coexist in an approximate 1:2 ratio with a free form, not crosslinked to the PG and surface-exposed^43^. Making Lpp longer could modify the ratio between the free and bound forms of Lpp, potentially affecting its contribution to cellular morphology.

Another important outcome of our work is that either removing Lpp from the envelope proteome or altering its structure strongly impacts cell envelope mechanics. Indeed, loss of Lpp, disruption of the covalent connection between this protein and the PG, and Lpp extension (by 7, 14, or 21 residues) all lead to a decrease in cell stiffness. These findings are of biological relevance because they reveal a novel function for Lpp in controlling the mechanics of the cell envelope and further highlight the crucial role played by this protein in maintaining the integrity of the OM, an essential permeability barrier that protects Gram-negative bacteria from a vast array of molecules with antibiotic properties. Clearly, the length and structural properties of Lpp have evolved in such a way that this protein is an important factor controlling envelope mechanics. In addition, our study shows that AFM-based mechanical experiments may contribute to the design of innovative antibacterial drugs that will disrupt the molecular machineries stabilizing the PG and OM layers, i.e., by targeting the Lpp-dependent cell wall properties.

Interestingly, disruption of the PG-OM covalent connection (Lpp^PG−^ and Lpp^+21,PG−^) or loss of Lpp leads to similar and significant cell softening, implying that covalent attachment of Lpp is the main factor contributing to cell stiffness, rather than the presence of the protein itself. Cells softening in cells with elongated Lpp, though more moderate, further highlights the importance of maintaining not only a covalent but also a space-controlled connection between the PG and the OM. The tiny differences, given the variability inherent to mid-exponential phase, in the mechanical behavior of elongated Lpp mutants might result from the number and density of Lpp-PG connections along the cell axis and at the poles. However, this question has not been thoroughly investigated yet, even for *E. coli* WT cells. This will require another study to tackle whether the mechanical role of Lpp is a matter of PG connections density or global OM–PG physical coupling. Moreover, a poorly understood question is whether Pal and OmpA connections also influence envelope mechanics. *E. coli* cells deleted for OmpA have been shown to be softer than WT cells^4^, but no AFM data are presently available for cells lacking Pal, the complex Tol-Pal being involved in the physical OM–PG coupling^4^. Clearly, further work on these proteins is needed to understand whether and how they orchestrate together with Lpp to regulate cell envelope mechanics.

Notably, vancomycin-treated cells are much softer when Lpp is artificially elongated or no longer covalently linked, implying that the Lpp-dependent mechanical properties of the cell envelope are of physiological importance. By contrast, the stiffness of WT cells and of cells lacking Lpp is not altered by the drug, suggesting therefore that the structural and chemical nature of the OM–PG connection is more important in defining drug susceptibility than the presence of Lpp per se. Intriguingly, while mutants lacking Lpp or the PG connection present the most prominent decrease in stiffness compared with the mutant with extended Lpp, in normal growth conditions, the latest shows the largest decrease in stiffness after treatment with vancomycin. We suggest that this peculiar phenotype may originate from the distance keeping function of Lpp. As specific envelope-spanning systems were shown to be potentially affected by Lpp extension^7,8^, it is tempting to speculate that envelope biogenesis machineries among others might be affected in the Lpp^+21^ mutant leading to higher vancomycin susceptibility. This could also explain why Lpp^+21,PG−^, after vancomycin treatment, behaves similarly to Lpp^+21^ rather than like Lpp^PG−^. Indeed, one would expect that when removing the physical coupling between the OM and the PG, further elongation of Lpp might not impact the OM properties. However, the presence of an elongated protein, expressed at high levels, in the interspace between the OM and the cell wall, might somehow destabilize the OM and, in turn increase its permeability.

## Methods

### Bacterial strains

We used seven *E. coli* strains, i.e., *E. coli* DH300 (referred to as WT)^44^, AA280, AA281, and AA282 expressing from the Lpp locus in DH300 an elongated version of Lpp by 7, 14, and 21 residues, respectively (in the text, Lpp^+7^, Lpp^+14^, and Lpp^+21^), AA261 expressing from the Lpp locus in DH300 an Lpp version lacking the terminal lysine residue and thus lacking the covalent bond of Lpp to PG (known as LppΔK58, but referred to in the text as Lpp^PG−^), Lpp^+21,PG−^, and AA132 lacking Lpp (ΔLpp) (Table 1). Details concerning the construction of *E. coli* Lpp mutants can be found in ref. ^7^. Lpp^+7^ was constructed in *E. coli* K12 F^−^ λ^−^ cells harboring the λ-Red recombineering plasmid (pKD46)^45^ as described previously^46^. Briefly, a tetracycline-resistance cassette (tetRA) was inserted between codons 42 and 43 of *E. coli* Lpp. Replacement of the tetRA cassette with insertions of 14 or 21 residues (two and three heptad repeats, respectively) was accomplished by introducing dsDNA fragments bearing 40 bp of homology to *E. coli* Lpp directly flanking the 5’ and 3’ ends of the tetRA cassette. Tetracycline-sensitive transformants (Lpp length mutants) were selected by plating on medium containing anhydrotetracycline (0.2 mg mL^−1^) and fusaric acid (2.4 mg mL^−1^). Chromosomal Lpp length mutations were moved into the MG1655 background through standard λ-Red recombineering as described in ref. ^47^. Primer sequences are available upon request. Lpp^+21,PG−^ mutant was constructed using site-directed mutagenesis of Lpp^+21^ chromosomal locus. It was amplified using primers: FW_AA116: ATACTTGTAACGCTACATGGAGATTAACTCAATCTAGAGGGTATTAATAATGAAAGCTACTAAACTGGTACTG.

**Table 1:** Strains used in this study.

Strains	Relevant genotype or features	Source or notes
DH300	rprA::lacZ MG1655 (argF-lac)U169 used as WT	Majdalani et al.^44^
AA132	DH300 ∆lpp	Asmar et al.^7^
AA261	DH300 Δlpp::lpp_∆K58_ referred to as lpp^PG−^	Asmar et al.^7^
AA280	DH300 Δlpp::lpp_+7_	This study
AA281	DH300 Δlpp::lpp_+14_	Asmar et al.^7^
AA282	DH300 Δlpp::lpp_+21_	Asmar et al.^7^
AA599	DH300 Δlpp::lpp_+21∆K58_, referred to as Lpp^+21PG−^	This study

REV_AA118: ACAAAAAAAATGGCGCACAATGTGCGCCATTTTTCACTTCACAGGTACTATTAGCGGTATTTAGTAGCCAT.

Then Lpp^+21,PG−^ was moved onto the chromosome of MG1655 cells via standard λ-Red recombineering as described in ref. ^47^.

### Bacterial growth conditions

Wild-type *E. coli* and its mutants were grown in LB broth overnight at 37 °C under continuous agitation. Fifty microliters of this solution was inoculated into 5 mL of prewarmed LB and cells were grown to mid-exponential growth phase (OD at 600 nm = 0.5–0.6) at 37 °C under stirring. Cells were centrifuged (6000 × *g* for 5 min) and washed twice by resuspension in PBS and centrifugation. Cells were diluted 1:10 in PBS. For vancomycin treatment, cells were grown to OD = 0.2, inoculated with a concentration of 100 µg mL^−1^ of vancomycin for 1 h 30 min and grown at 37 °C under continuous agitation.

### Phase-contrast microscopy

Strains were grown to an OD_600_ of 0.5. One milliliter of culture was collected, resuspended in same volume PBS, and kept for 2 h at room temperature to recapitulate the AFM imaging conditions. Then cells were mixed with 50 µL of 10× fixing solution (0.4% glutaraldehyde, 25% formaldehyde, 330 mM sodium phosphate [pH 7.6]) for 30 min at room temperature. Cells were spun down at 6000 × *g* at room temperature, resuspended in 50 µL PBS, and imaged on agarose pads. Fixed samples were imaged with an Axio Observer Z1 inverted epifluorescence microscope (Carl Zeiss) equipped with an Axiocam 506 mono camera (Carl Zeiss), 1×/C-mount camera adapter (Carl Zeiss), phase-contrast objective Plan Apochromat 100×/1.40 Oil Ph3 (Carl Zeiss). Images were acquired with Zen 2 (blue edition; Carl Zeiss). Exposure times and image scaling were identical for compared conditions. MicrobeTracker^48^ was used to obtain cell outlines. Quantitative analysis from cell meshes was done with MATLAB R2014a (Mathworks, Inc.) using custom scripts to plot the distributions of lengths widths and estimated volumes for the strains tested.

### Growth curves

Single colonies were used to inoculate overnight cultures, which were diluted 1:100 or 1:200, in round-bottom 96-well plates in 200 µL LB. Absorbance was measured at 600 nm every 15 min in a Synergy H1 microplate reader (BioTek) with constant orbital shaking at 37 °C.

### AFM multiparametric imaging

AFM images and force–distance curves were recorded in PBS at room temperature using a NanoWizard III AFM (JPK Instruments). To immobilize bacteria, we used polyethylenimine (PEI)-coated glass coverslips. To this end, 100 µL of PEI 0.2% were dropped on a glass coverslip, left overnight at room temperature, rinsed heavily with Milli-Q water, and dried with a nitrogen flow. One hundred microliters of the bacterial diluted suspension were then incubated for 1 h on this chemically functionalized substrate and rinsed three times in PBS bath before any AFM measurements. Though a nutrient medium (like LB) might be more adapted for bacteria metabolism, this medium prevents cells from adhering to the PEI-coated coverslips, their division, and swimming being more favorable in such medium. Consequently, PBS was chosen as the best solvent for our experiments, after also making sure that cell morphology and mechanics were not dependent on the time spent in PBS (Supplementary Fig. 3). Imaging was performed in the QI mode with MSCT cantilevers, whose spring constants ranged from 0.17 to 0.22 N m^−1^ (thermal noise method). Images and force curves were recorded at a scan rate of 25 µm/s, an applied force of 0.25 nN, and with a resolution of 128 × 128 pixels^2^. Data were analyzed offline using the JPK data processing software. For mechanical analysis, the approach part of the curves was fitted with the Hertz model (Poisson ratio, *ν* = 0.5) over a distance of 20 nm and considering a conical tip (opening angle of 17.5°)^49^.

### Mechanical study by AFM FV analysis

We also recorded force–distance curves in the FV mode. Curves were recorded with a maximum applied force of 0.5 nN, approach and retraction velocities of 1 µm s^−1^, and a 0.5 µm ramp size. For each cell, a map of 16 × 16 curves was recorded on a 250 × 250 nm² area on top of the cell, in the central region to avoid edge effects. Similar data processing as for QI measurements were performed on these FV experiments. In addition to the Young’s modulus extracted in the nonlinear region of the curves, from the Hertz model, the spring constant over the linear part was also determined here. Also, the cell indentation is presented in Supplementary Fig. 8.

### MIC determination

MIC values were determined using VANCOMYCIN VA 256 WW S30 Etest^®^ from Biomerieux^®^. Cells were grown to an OD_600_ = 0.6. Then 200 µL of culture were mixed with 4 mL top agar and spread on LB plates. The strips were put on solidified top agar then incubated at 37 °C overnight. MIC values were determined as the intersection between the inhibition ellipse and the edge of the strip.

### Vancomycin sensitivity spotting assay

Cells were grown to exponential phase OD_600nm_ = 0.5, then serially diluted by tenfold in LB up to 10^−7^. Two microliters of each dilution were spotted on LB agar overnight at 37 °C. When indicated, vancomycin was added to the LB agar to a final concentration of 100 µg mL^−1^.

### Statistical methods

The statistical significance of differences among bacterial strains was assessed using GraphPad Prism 8 according to analysis of variance followed by the application of Tukey’s multiple-comparison test when the distribution was normal. Otherwise, the Kruskal–Wallis test was used followed by Dunn’s multiple-comparison test. Normality was assessed using the Shapiro–Wilk test. *P* values when differences are significant are provided on graphs and in figure legends.

### Reporting summary

Further information on research design is available in the Nature Research Reporting Summary linked to this article.

## Supplementary information

Supplementary Information

Reporting Summary

## Data Availability

The Source Data underlying Figs. 3–6 and Supplementary Figs. 1–8 are provided as a separate Source Data file. All other relevant data are available from the corresponding author upon request.

## Source Data

Source Data
