# Supplementary material for: Lipoprotein Lpp regulates the mechanical properties of the E. coli cell envelope
Source: Nat Commun. 2020 Apr 14;11:1789. doi: 10.1038/s41467-020-15489-1 (PMC7156740; doi:10.1038/s41467-020-15489-1)
Supplement: Supplementary file 1 — Supplementary Information [file 41467_2020_15489_MOESM1_ESM.pdf]

Supplementary Information for

Lipoprotein Lpp regulates the mechanical properties of the E. coli cell envelope

Mathelié-Guinlet *et al.*

This PDF file includes supplementary figures 1 to 8.

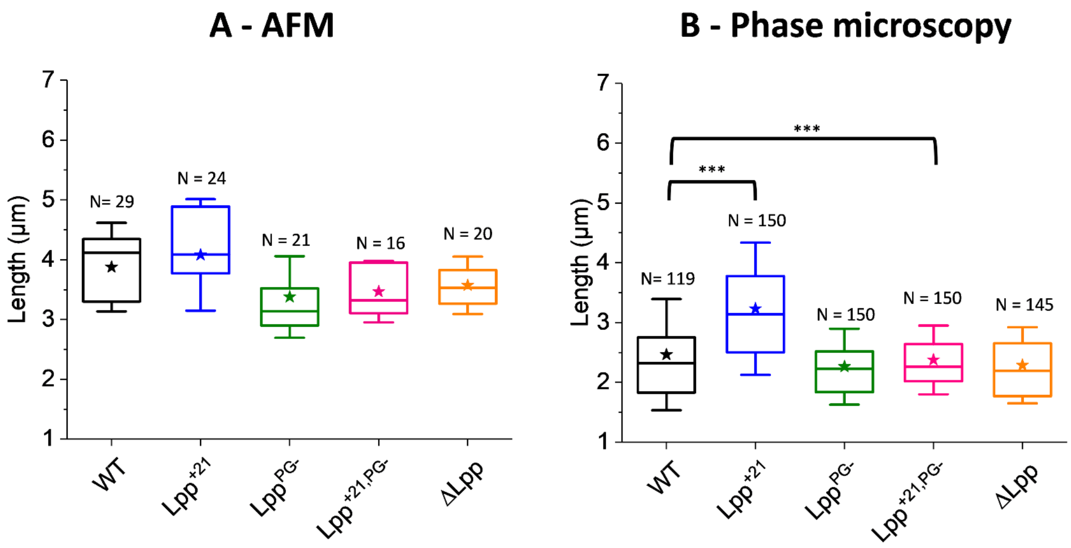

Supplementary Figure 1. Effect of Lpp mutations on *E. coli* length. Box plots reporting the length of each bacterial strain estimated from (A) AFM images and (B) phase microscopy cliches. Shown here are the mean values (star), the median, the 25% and 75% quartiles (boxes), and the standard deviation (whiskers) obtained from N independent cells over at least three independent experiments. *** indicates $P \leq 0.0001$. Source data are provided as a Source Data file.

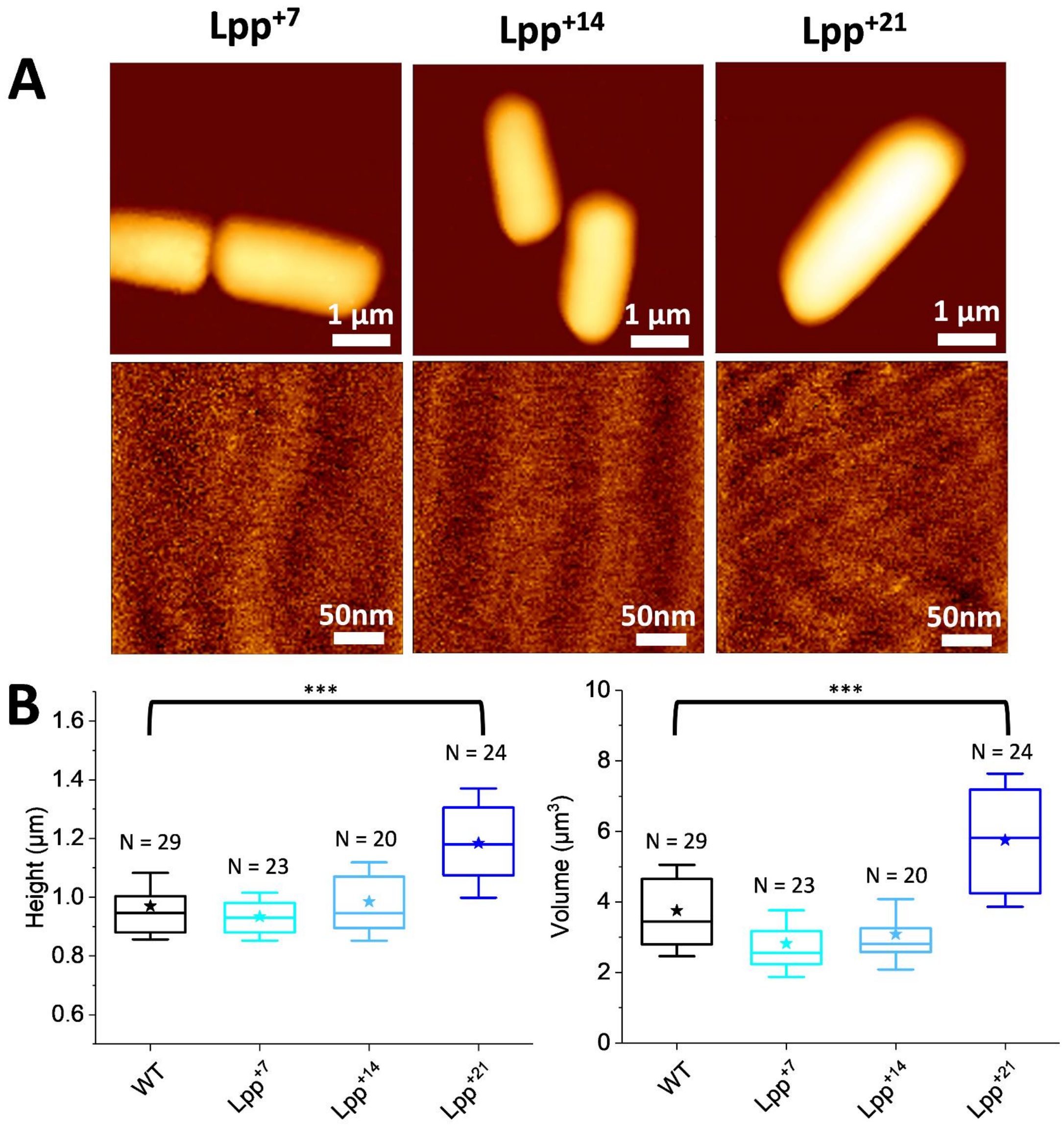

Supplementary Figure 2. Effect of Lpp length on *E. coli* morphology. (A) AFM height images in PBS showing *E. coli* cells with mutations on the length of Lpp (color scale: 1.2 µm). High-resolution height images recorded on top of the cells are also shown (color scale: 10 nm). These images are representative of at least three independent experiments. (B) Box plots reporting the height and volume of each bacterial strain exhibiting a longer version of Lpp, estimated from AFM images. Shown here are the mean values (star), the median, the 25% and 75% quartiles (boxes), and the standard deviation (whiskers) obtained from N independent cells over at least three independent experiments. *** indicates $P \leq 0.0001$. Source data are provided as a Source Data file.

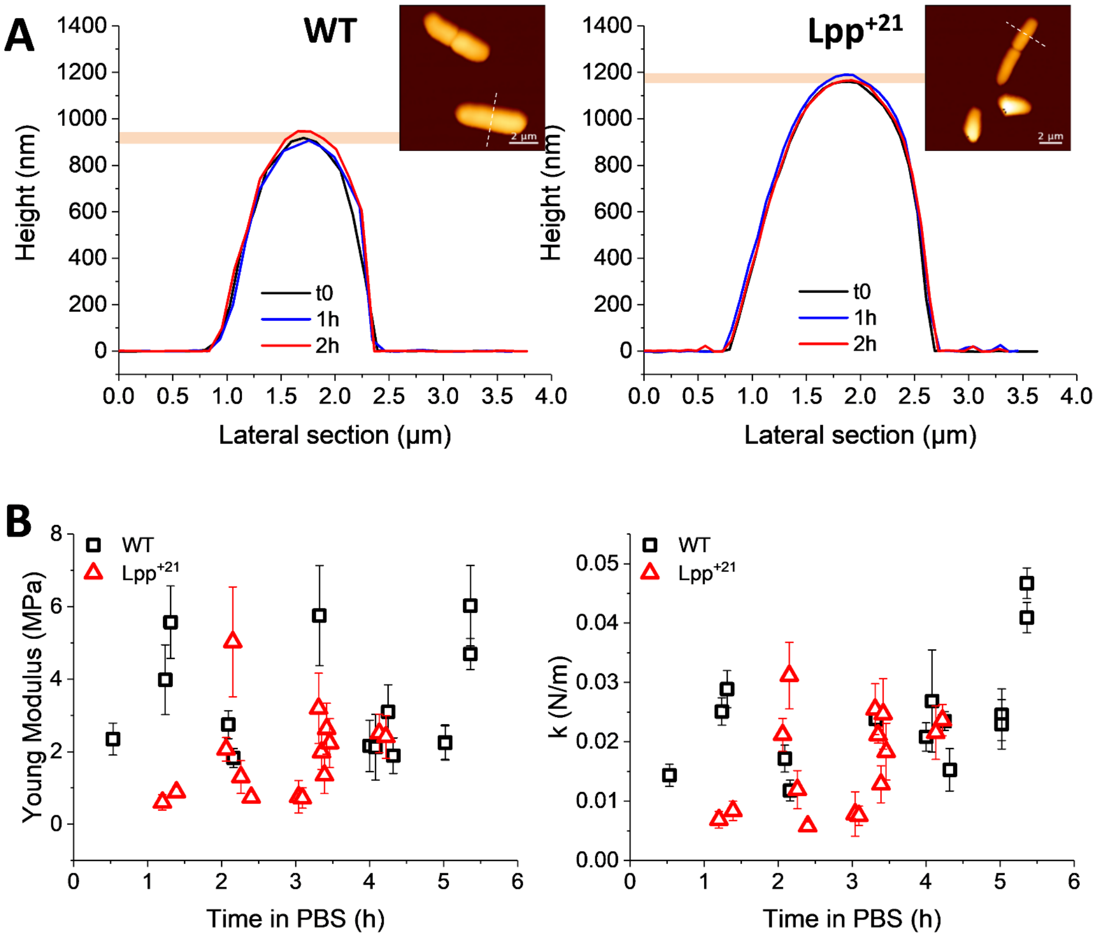

Supplementary Figure 3. PBS has no influence on the morphology or mechanics of *E. coli* cells. (A) Height profiles of the same cell (left – WT, right – Lpp^{+21}) imaged in PBS over 2 h, showing no substantial changes in cell height. This is representative of at least three independent cells. (B) Mechanical properties of WT and Lpp^{+21} cells as a function of the time spent in PBS, also showing no significant effect of PBS. Each dot represents one independent cell (mean ± SD). Source data are provided as a Source Data file.

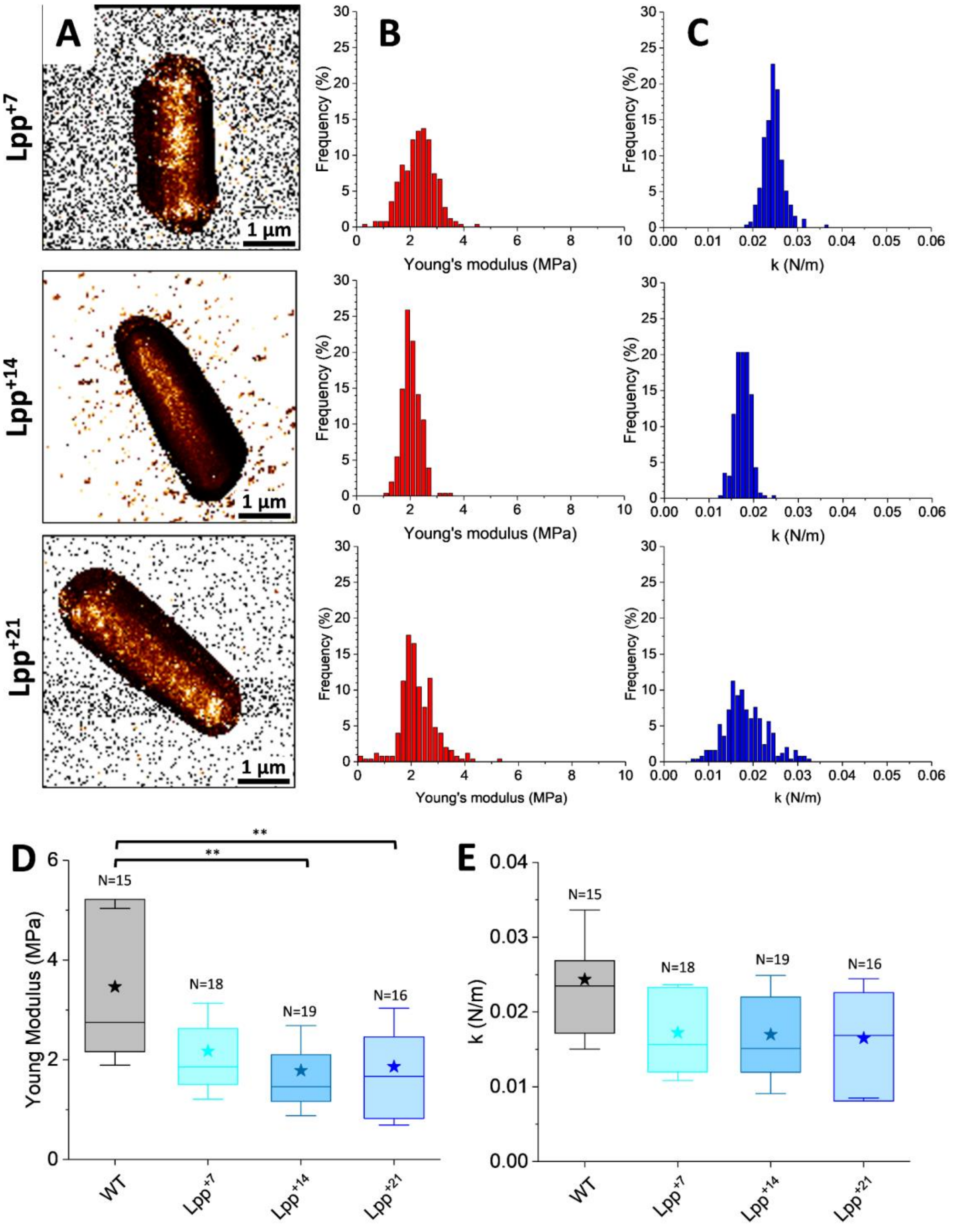

Supplementary Figure 4. Effect of Lpp length on *E. coli* cell envelope mechanics. (A) Elasticity maps in PBS of representative cells from the three strains exhibiting a longer version of Lpp (QI mode, 128 x 128 curves, color scale: 10 MPa). These images are representative of at least three independent experiments. Distribution of Young's modulus (B) and spring constant (C) values obtained by force-volume measurements across the surface of one representative cell (16 x 16 curves, 250 x 250 nm²) of each strain. Statistical analysis performed for each strain, showing the Lpp length-dependent softening of *E. coli* strains: (D) Young's modulus and (E) spring constant. Shown here are the mean values (star), the median, the 25% and 75% quartiles (boxes), and the standard deviation (whiskers) obtained from N independent cells over at least three independent experiments. ** indicates $P \leq 0.001$. Source data are provided as a Source Data file.

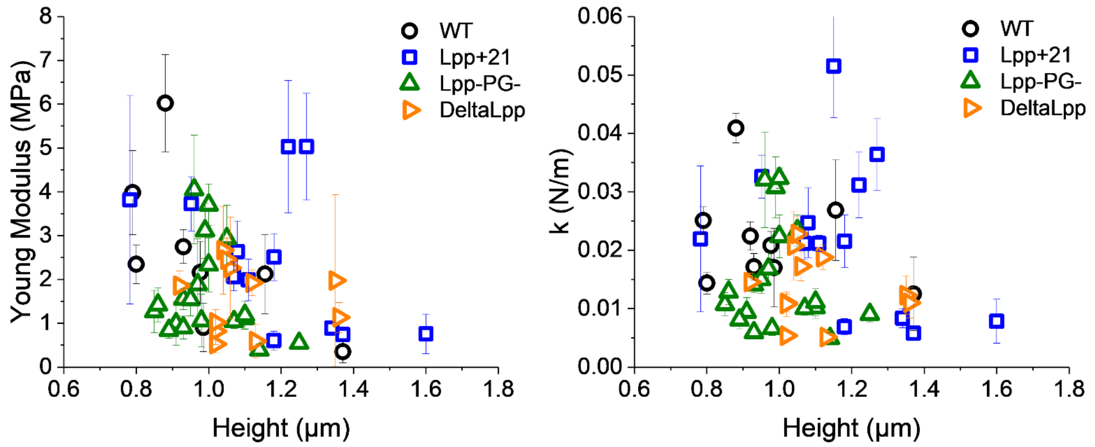

Supplementary Figure 5. Morphological-dependent mechanical properties of *E. coli*. Young's modulus and spring constant of some representative cells for each of the main Lpp mutation as a function of cell height. Each dot represents one independent cell (mean ± SD). Source data are provided as a Source Data file.

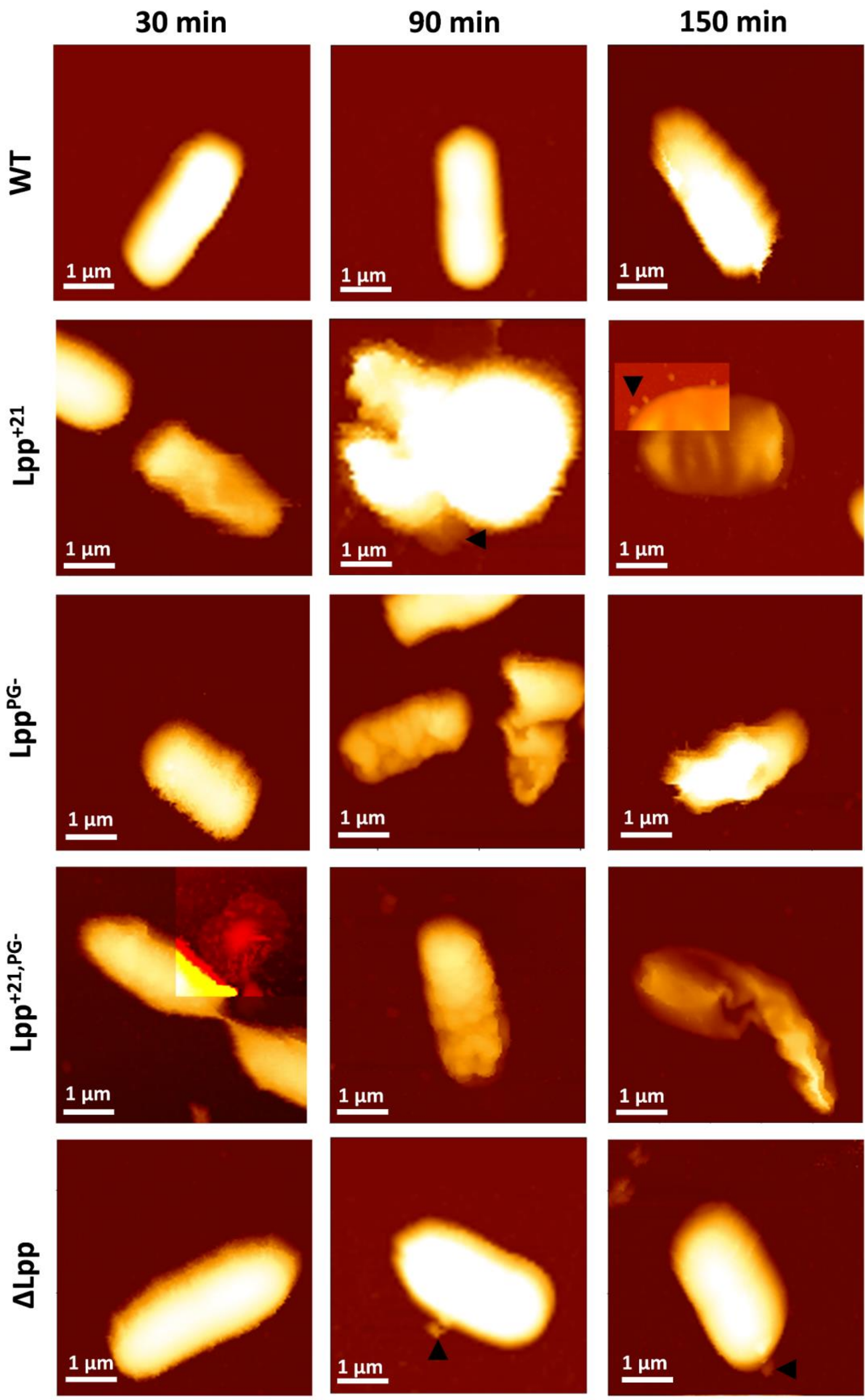

Supplementary Figure 6. Vancomycin treatment damages the morphology of *E. coli* cells in a time and Lpp-mutation dependent manner. AFM height images (color scale: 1.2 μm) of *E. coli* strains after vancomycin treatment over time (100 μg/mL). Interesting features are highlighted with black arrows. These images are representative of at least three independent experiments.

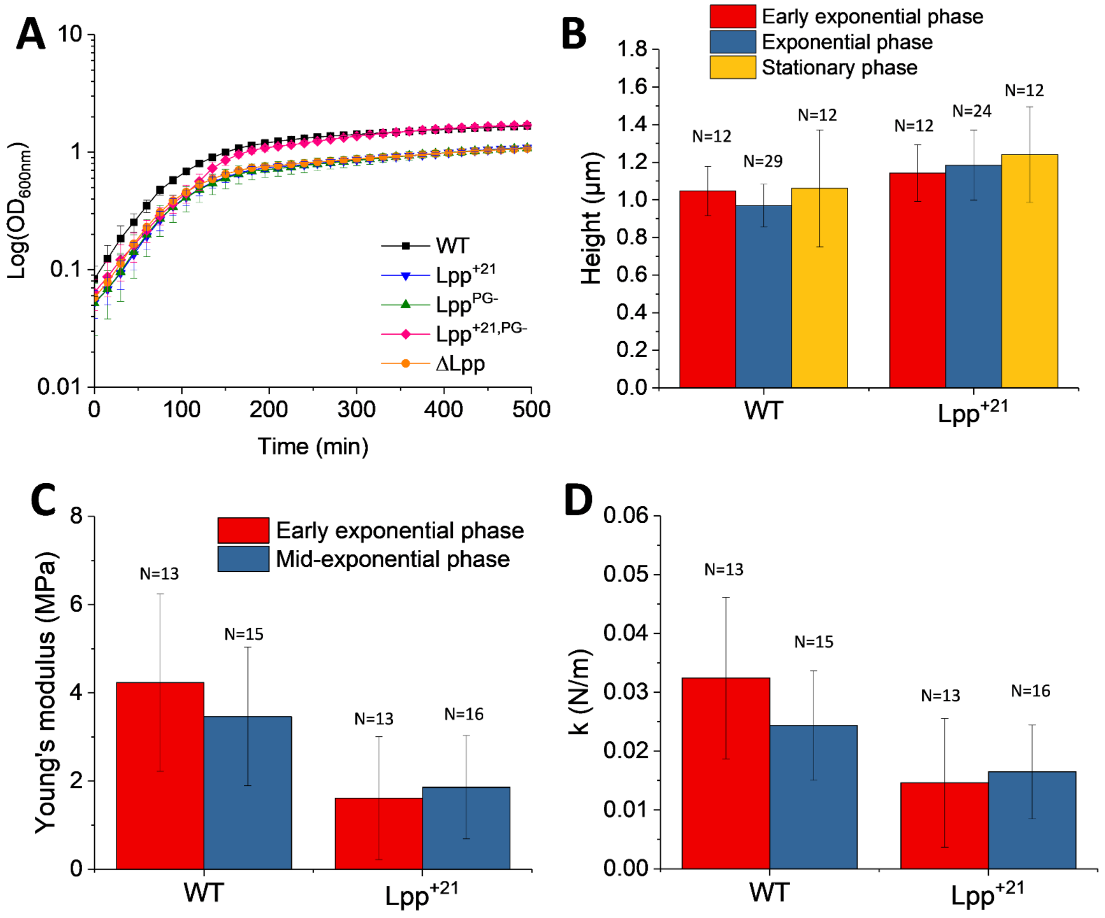

Supplementary Figure 7. Effect of Lpp mutations on *E. coli* growth rate. (A) Instantaneous growth rate of *E.coli* cells depending on the Lpp mutation. Error bars account for three replicates. (B) Influence of the growth state of WT and Lpp^{+21} cells on their height (data are presented as mean ± SD over N independent cells), estimated from AFM images. (C-D) Influence of the growth state of WT and Lpp^{+21} cells on their mechanical properties, Young's modulus and spring constant (data are presented as mean ± SD over N independent cells). The early exponential phase corresponds to an OD_{600} of 0.2-0.3, the mid-exponential phase to an OD_{600} of 0.5-0.6 and the stationary phase to an OD_{600} of 1.0-1.2. Source data are provided as a Source Data file.

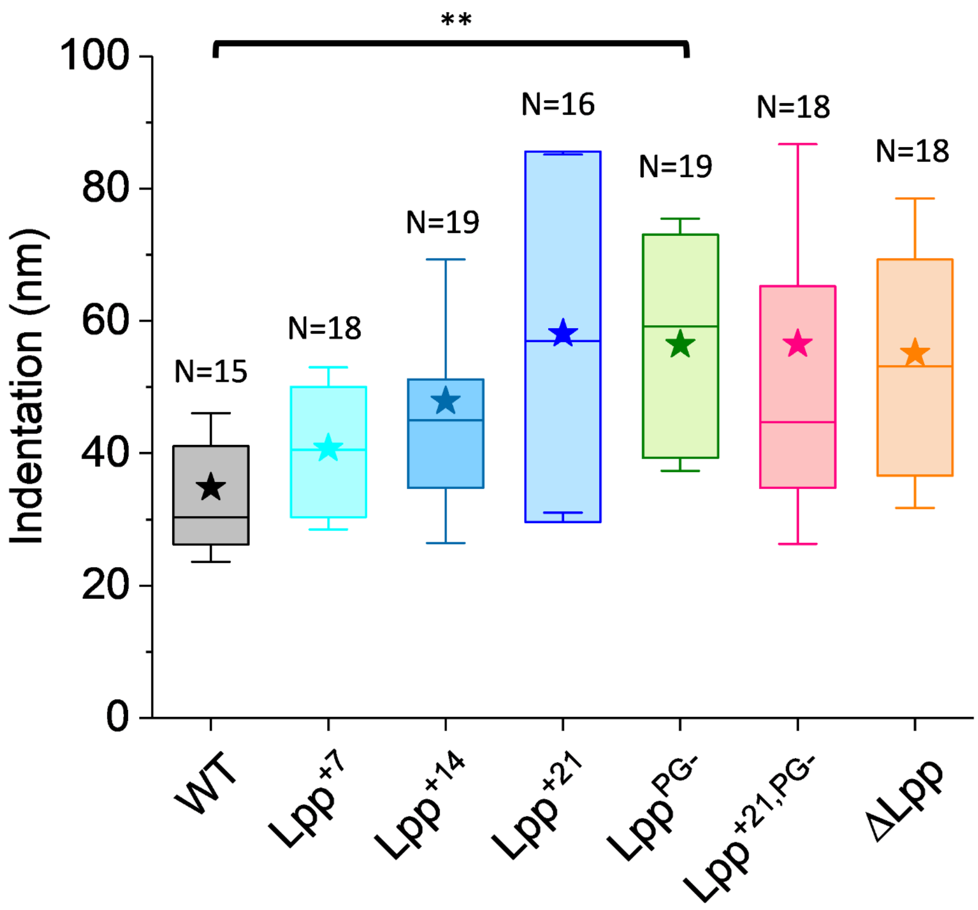

Supplementary Figure 8. *E. coli* cell deformation depends on the Lpp functional mutation. Box plot reporting the indentation observed on each bacterial strain when recording force curves at F = 0.5 nN. Shown here are the mean values (star), the median, the 25% and 75% quartiles (boxes), and the standard deviation (whiskers) obtained from N independent cells over at least three independent experiments. ** indicates $P \leq 0.001$. Source data are provided as a Source Data file.
